# Reciprocal Allosteric Modulation of Carbon Monoxide and Warfarin Binding to Ferrous Human Serum Heme-Albumin

**DOI:** 10.1371/journal.pone.0058842

**Published:** 2013-03-21

**Authors:** Alessio Bocedi, Giampiero De Sanctis, Chiara Ciaccio, Grazia R. Tundo, Alessandra Di Masi, Gabriella Fanali, Francesco P. Nicoletti, Mauro Fasano, Giulietta Smulevich, Paolo Ascenzi, Massimo Coletta

**Affiliations:** 1 Department of Clinical Sciences and Translational Medicine, University of Roma Tor Vergata, Roma, Italy; 2 Department of Molecular, Cellular and Animal Biology, University of Camerino, Camerino (MC), Italy; 3 Interuniversity Consortium for the Research on the Chemistry of Metals in Biological Systems, Bari, Italy; 4 Department of Biology and Interdepartmental Laboratory for Electron Microscopy, University Roma Tre, Roma, Italy; 5 Department of Structural and Functional Biology and Center of Neuroscience, University of Insubria, Busto Arsizio (VA), Italy; 6 Department of Chemistry “Ugo Schiff”, University of Firenze, Sesto Fiorentino (FI), Italy; Aligarh Muslim University, India

## Abstract

Human serum albumin (HSA), the most abundant protein in human plasma, could be considered as a prototypic monomeric allosteric protein, since the ligand-dependent conformational adaptability of HSA spreads beyond the immediate proximity of the binding site(s). As a matter of fact, HSA is a major transport protein in the bloodstream and the regulation of the functional allosteric interrelationships between the different binding sites represents a fundamental information for the knowledge of its transport function. Here, kinetics and thermodynamics of the allosteric modulation: (i) of carbon monoxide (CO) binding to ferrous human serum heme-albumin (HSA-heme-Fe(II)) by warfarin (WF), and (ii) of WF binding to HSA-heme-Fe(II) by CO are reported. All data were obtained at pH 7.0 and 25°C. Kinetics of CO and WF binding to the FA1 and FA7 sites of HSA-heme-Fe(II), respectively, follows a multi-exponential behavior (with the same relative percentage for the two ligands). This can be accounted for by the existence of multiple conformations and/or heme-protein axial coordination forms of HSA-heme-Fe(II). The HSA-heme-Fe(II) populations have been characterized by resonance Raman spectroscopy, indicating the coexistence of different species characterized by four-, five- and six-coordination of the heme-Fe atom. As a whole, these results suggest that: (i) upon CO binding a conformational change of HSA-heme-Fe(II) takes place (likely reflecting the displacement of an endogenous ligand by CO), and (ii) CO and/or WF binding brings about a ligand-dependent variation of the HSA-heme-Fe(II) population distribution of the various coordinating species. The detailed thermodynamic and kinetic analysis here reported allows a quantitative description of the mutual allosteric effect of CO and WF binding to HSA-heme-Fe(II).

## Introduction

Human serum albumin (HSA), the most abundant protein in human plasma (with a bloodstream concentration of about 0.7 mM), binds a wide variety of endogenous ligands including non-esterified fatty acids, bilirubin, hemin and hormones. HSA also binds exogenous ligands, such as drugs, undertaking their distribution and delivery, thus representing an important determinant of pharmacokinetics. Physiological or pathological conditions that induce variations in the plasma level of HSA as well as genetic polymorphisms of HSA could alter exogenous and endogenous ligand binding, compromising their transport and metabolism [1]–[3].

**Figure 1:**
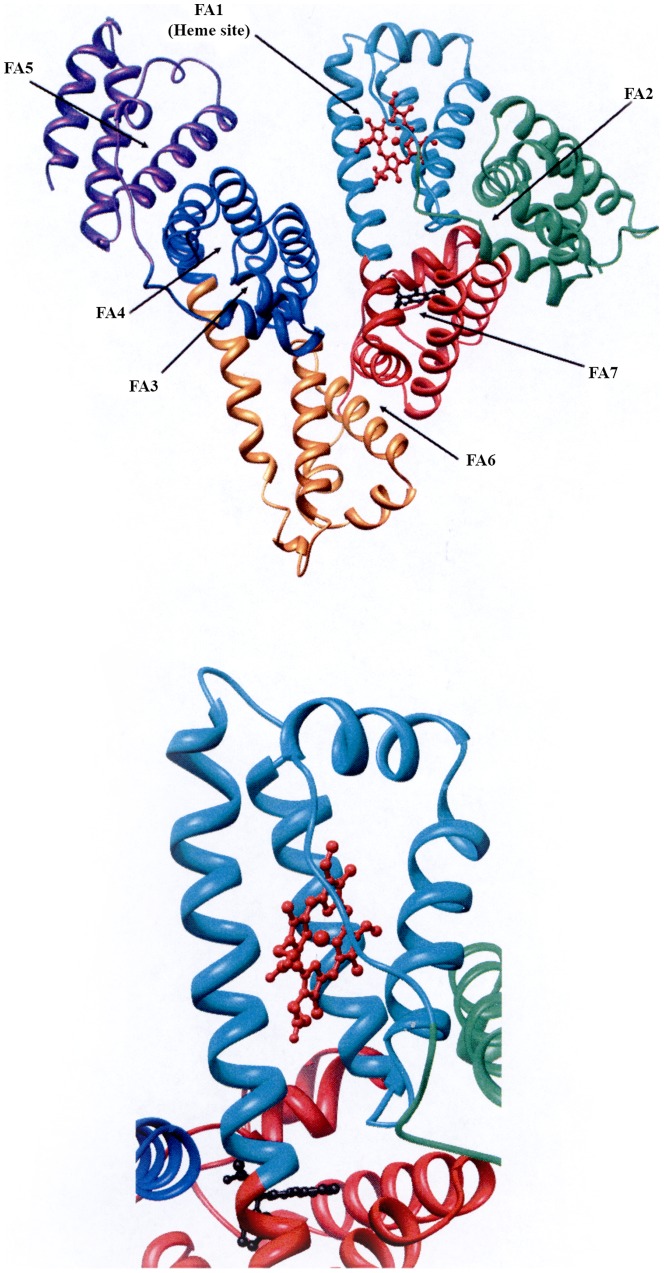
HSA Structure. (A) Ribbon representation of HSA-heme-Fe(III) The six subdomains of HSA are coloured as follows: subdomain IA, green; subdomain IB, cyan; subdomain IIA, red; subdomain IIB, orange; subdomain IIIA, blue; and subdomain IIIB, purple. (B) Enlarged view of allosteric FA1 and FA7 heme-Fe(III) sites. Heme-Fe(III) (in red) and WF (in black) are rendered as balls and sticks. Atomic coordinates were taken from PDB entries 1O9X [14] and 2BXD [29]. The structural model was drawn with the UCSF Chimera package.

HSA is a monomer composed by 585 amino acid residues ordered in three homologous α-helical domains (I–III); each domain is divided into the A and B subdomains connected by random coils. Terminal regions of sequential domains contribute to the formation of interdomain α-helices linking subdomains IB to IIA, and IIB to IIIA (Fig. 1A) [1], [3], [4]–[18].

The structural organization of HSA provides a variety of functionally linked binding sites. In particular, the heme binds with high affinity (*K_d_* ∼ 1×10^−8^ M) [19], [20] within a narrow oblate hydrophobic cavity in the HSA subdomain IB [12], [14], [21], which is called fatty acid binding site 1 (FA1; see Fig. 1A) and represents the “heme binding cleft”. This cavity is limited by Tyr138 and Tyr161 residues that provide π-π stacking interactions with the porphyrin and supply a donor oxygen (from Tyr161) to the heme-Fe(III)-atom for the formation of human serum heme-albumin (HSA-heme) [3], [12], [14], [19], [21]–[23]. Further, two main drug-binding pockets are Sudlow’s site I (located in subdomain IIA and corresponding to the FA3-FA4 cleft) and Sudlow’s site II (located in subdomain IIIA and corresponding to the FA7 site). Warfarin (WF) and ibuprofen (IBU) are considered as the stereotype ligands for the FA7 and the FA3-FA4 cleft, respectively. However, ibuprofen binds also to the FA2 and FA6 secondary sites, moreover FA2 has been postulated to be the secondary site of WF [1], [3], [6], [10], [11], [24]–[29]. Therefore, the occurrence of multiple binding pockets allows an allosteric behaviour of HSA due to the fact that the ligand occupancy of one (or more) sites(s) brings about an alteration of the functional properties of the other ones. Thus, the heme-binding cleft (i.e., FA1) and Sudlow’s site I (i.e., FA7) are allosterically coupled (Fig. 1B), since the heme-Fe(III) affinity for HSA decreases by about one order of magnitude upon WF binding; accordingly, heme-Fe(III) binding to HSA decreases the affinity of ligands (e.g., drugs) for Sudlow’s site I by the same extent [3], [17], [19]–[21], [30]–[35].

Ferrous HSA-heme (HSA-heme-Fe(II)) binds nitrogen monoxide (NO) and carbon monoxide (CO) and exhibits a weak catalase and peroxidase activity, involving the heme-Fe complex in the maintenance of antioxidative homeostasis in the extracellular fluids [3], [8], [13], [26], [36]–[44]. Remarkably, HSA-heme-Fe(II) reactivity has been reported to be modulated allosterically by drugs, indeed abacavir facilitates peroxynitrite-mediated oxidation of ferrous nitrosylated HSA (HSA-heme-Fe(II)-NO), in the absence and presence of CO_2_ [42]. Moreover, abacavir, IBU and WF facilitate NO dissociation from HSA-heme-Fe(II)-NO [43], [44].

Kinetics of CO binding to HSA-heme-Fe(II) and CO dissociation from ferrous carbonylated HSA-heme (HSA-heme-Fe(II)-CO) in the absence of third components displays a multiphasic reaction pattern which suggests that various heme-protein coordination forms may coexist in a slow equilibrium among each other [45]. Here, the effect of the prototypical drug WF on CO binding to HSA-heme-Fe(II) and CO dissociation from HSA-heme-Fe(II)-CO and vice versa is reported and a similar behavior is observed, even though rates and relative percentages of the multiphasic reaction pattern are affected by WF. In parallel, WF binding to HSA-heme-Fe(II), in the absence and presence of saturating CO concentration shows as well multiple exponentials. The HSA-heme-Fe(II) populations have been characterized by resonance Raman (RR) spectroscopy, indicating the coexistence of different species characterized by four-, five- and six-coordination of the heme-Fe atom. These results highlight the allosteric modulation of HSA-heme reactivity by heterotropic interaction(s), and outline the role of drugs in modulating HSA functions. This work represents a detailed kinetic and thermodynamic study on the allosteric interaction, employing a drug as an heterotropic allosteric effector also to outline the important role of HSA as a drug carrier and the importance of allostery for this function. Therefore, it represents a very important and widespread mechanism of action, where allostery works for drug uptake and delivery, representing the main mechanism regulating the transport function of HSA.

## Materials and Methods

### Materials

All reagents were obtained from Sigma-Aldrich (St. Louis, MO, USA) with the exception of CO that was purchased from Linde AG (Höllriegelskreuth, Germany) or Rivoira (Milan, Italy), and ^13^CO that was purchased from FluoroChem (Hadfield, UK). All chemicals were of the highest purity available and were used without further purification.

HSA (from Sigma-Aldrich) was FA-free according to the charcoal delipidation protocol [46]-[48] and its concentration was determined by the Bradford assay [49]. Ferric HSA-heme (HSA-heme-Fe(III)) was prepared by adding the appropriate volume of the heme-Fe(III) stock solution (i.e., 1.2×10^−2^ M heme-Fe(III) in 1.0×10^−1^ M NaOH) to the 1.0×10^−3^ M HSA stock solution (in 1.0×10^−1^ M phosphate buffer, pH 7.0) in order to obtain a final HSA-heme-Fe(III) concentration of about 1.0×10^−5^ M. Heme-Fe(III) binding to HSA was monitored spectrophotometrically using an optical cell with 1.0-cm path length on a Jasco V-53 spectrophotometer (Varian Inc., Palo Alto, USA) in the UV-Vis region (300–800 nm). The HSA:heme-Fe(III) ratio was about 10∶6 and the excess of HSA ensured that the heme-Fe(III) was bound only to the FA1 site and no free heme-Fe(III) was present in solution [28]. The heme-Fe(III) concentration was determined spectrophotometrically at 535 nm after converting hemin to the heme-Fe(III)-bis-imidazolate derivative in sodium dodecylsulfate micelles (ε_535_  =  14.5 cm^−1^ mM^−1^) [50], [51]. HSA-heme-Fe(II) was obtained by adding sodium dithionite (final concentration about 5 mg/ml) to the HSA-heme-Fe(III) solution [52].

The CO stock solution was prepared by keeping anaerobically in a closed vessel distilled water under CO at P  =  760.0 mm Hg (T  =  20°C). The solubility of CO in water is 1.03×10^−3^ M, at P  =  760.0 mm Hg and 20°C [52].

The WF stock solution ( =  5.0×10^−2^ M) was prepared by dissolving the drug in 1.0×10^−1^ M phosphate buffer, pH 7.0 and acetone 20%; acetone (final concentration ≤ 4%) does not affect spectroscopic properties of the solutions and HSA-heme-Fe(II) reactivity.

HSA-heme-Fe(II)-CO and warfarin-bound ferrous HSA-heme (WF-HSA-heme-Fe(II)) solutions were prepared by equilibrating for 1 hr a HSA-heme-Fe(III) solution (about 1.0×10^−5^ M), in the presence of 5 mg/ml of sodium dithionite, with either 1.0×10^−4^ M CO or 1.0×10^−2^ M WF. The unusually-long equilibration time was necessary for the completion of the reactions, which are characterized by very slow phases (see Results).

For the RR experiments, HSA-heme-Fe(II)-CO, in the absence and presence of WF, was prepared by degassing the HSA-heme-Fe(III) solution by flushing first with nitrogen and then with CO or ^13^CO and reducing the heme by addition of a 5% volume freshly prepared sodium dithionite (20 mg/mL) solution; the final heme-Fe(III):WF molar ratio was 1:1000.

All the experiments have been carried out at pH 7.0 (1.0×10^−3^ M phosphate buffer and 1.0×10^−1^ M phosphate buffer) and 25°C.

## Methods

### Thermodynamics of WF binding to HSA-heme-Fe(II)

**Figure 2:**
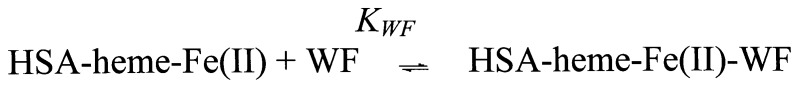
Scheme for the WF equilibrium binding to HSA-heme-Fe(II).



The value of the association equilibrium constant for WF binding to HSA-heme-Fe(II) (i.e., *K_WF_*) was determined spectrophotometrically (between 380 and 480 nm) by stepwise addition of the WF stock solution to the HSA-heme-Fe(II) or HSA-heme-Fe(II)-CO samples and measuring the variations of optical density as a function of the WF concentration (i.e., [WF]), employing a double-beam Cary 5 spectrophotometer (Varian, Palo Alto, Ca, USA). Data were analyzed in the framework of the minimum reaction mechanism depicted in Figure 2 according to eq. (1),

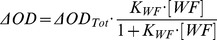
(1)where *ΔOD* is the measured optical density change at 414 nm with respect to the sample without WF, *ΔOD_Tot_* is the total optical density change observed when the sample is saturated with WF [45].

### Kinetics of WF and CO binding to HSA-heme-Fe(II)

Kinetic progress curves for CO binding to HSA-heme-Fe(II), in the presence of WF, and for WF binding to HSA-heme-Fe(II), in the absence and presence of CO, have been obtained employing a SX.18MV stopped-flow apparatus provided with the diode array accessory for transient spectra collection (Applied Photophysics, Salisbury, UK). Absorbance spectra have been collected between 380 and 700 nm with a 1.5 ms time resolution.

CO and/or WF association kinetics have been undertaken by mixing: (i) WF-HSA-heme-Fe(II) with CO (final concentration ranging between 2.0×10^−5^ M and 4.0×10^−4^ M) solution, (ii) HSA-heme-Fe(II) with WF (final concentration ranging between 5×10^−5^ M and 1.0×10^−2^ M) solution, and (iii) HSA-heme-Fe(II)-CO with WF (final concentration ranging between 5×10^−5^ M and 1.0×10^−2^ M) solution. Kinetics of HSA-heme-Fe(II) carbonylation in the absence of WF has been previously reported [45].

Values of the first-order rate constant for CO dissociation from HSA-heme-Fe(II)-CO, in the presence of 1.0×10^−2^ M WF (i.e., *k_offWFCO_*) were obtained by rapid mixing the protein solution(s) with the sodium nitrite (final concentration 5.0×10^−3^ M) solution in the presence of sodium dithionite (5.0 mg/mL final concentration). The reaction of sodium dithionite with sodium nitrite induces a very rapid production of NO; CO replacement by NO is rate-limited by CO dissociation [45], [52]. Kinetics of HSA-heme-Fe(II)-CO decarbonylation in the absence of WF (i.e., *k_offCO_*) has been previously reported [45].

All kinetic progress curves have been analyzed according to the following equation:
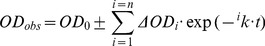
(2)where *OD_obs_* is the observed optical density at 414 nm at a given time interval, *OD_0_* is the optical density at *t*  =  0, *n* is the number of exponentials, *ΔOD_i_* is the optical density change associated to the exponential *i*, *^i^k* is the rate constant of the exponential *i* (referring to either the ligand binding *^i^k_obs_* or the ligand dissociation *^i^k_off_*) and *t* is the time [45].

Three types of rate constants for the association of either CO and/or WF to HSA-heme-Fe(II) have been observed. Namely: (i) rate constants which are linearly dependent on the ligand (i.e., CO or WF) concentration, displaying a bimolecular behaviour, (ii) rate constants, whose ligand concentration dependence is not linear, showing a rate-limiting step behaviour, and (iii) rate constants which are independent on the ligand concentration.

Rate constants displaying a bimolecular behaviour have been analyzed according to the following equation:



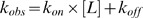
(3)where *k_obs_* is the observed rate constant, as obtained from the analysis of the kinetic progress curve according to the Eq. (2), *k_on_* is the second-order association rate constant for the ligand *L* (i.e., either CO or WF), *k_off_* is the first-order ligand dissociation rate constant, and [*L*] is the ligand concentration (either CO or WF) [45].

**Figure 3:**
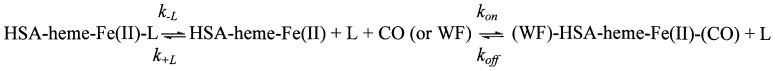
Scheme for the CO (and/or WF) binding to the endogenously hexa-coordinated HSA-heme-Fe(II)-L species.



Rate constants displaying a rate-limiting behaviour have been described by the minimum reaction reported in Figure 3 [53], where *L* is an endogenous ligand (likely a protein residue coordinating the heme), which must dissociate before an exogenous ligand (i.e., CO or WF) can bind. In the case of CO as the external ligand, the observed rate constant for the formation of the final HSA-heme-Fe(II)-(CO) complex (i.e., *k_obs_*) has been described by eq. (4a):

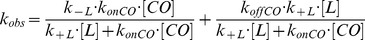
(4a)Under conditions where the concentration of the endogenous ligand [*L*] is constant and *k_+L_’*  =  *k_+L_* × [*L*], eq. (4a) reduces to eq. (4b) [53]:
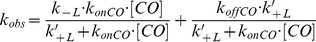
(4b)


Obviously, the equations 4a and 4b apply also in the case of WF as the exogenous ligand.

Calculation of kinetic and thermodynamic parameters has been undertaken, employing MatLab version 7.0.(Mathworks, USA).

### Spectroscopy of HSA-heme-Fe(II)-CO

Electronic absorption spectra of HSA-heme-Fe(II)-CO, in the absence and presence of WF, were collected with a double-beam Cary 5 spectrophotometer (Varian, Palo Alto, CA) using a 5-mm NMR tube or a 1-mm cuvette, and a 600 nm/min scan rate. RR spectra were obtained using a 5-mm NMR tube and by excitation with the 413.1 nm line of a Kr^+^ laser (Coherent, Innova 300 C, Santa Clara, CA) and the 441.6 nm line of a HeCd laser (Kimmon IK4121RG, Tokyo Japan). Backscattered light from a slowly rotating NMR tube was collected and focused into a triple spectrometer as previously reported [23]. All RR measurements were repeated several times under the same conditions to ensure reproducibility. To improve the signal/noise ratio, a number of spectra were accumulated and summed only if no spectral differences were noted. The RR spectra were calibrated with indene, CCl_4_, dimethyl sulfoxide, acetone, and acetonitrile as standards to an accuracy of 1 cm^-1^ for intense isolated bands.

## Results

### Thermodynamics of WF binding to HSA-heme-Fe(II)

**Figure 4:**
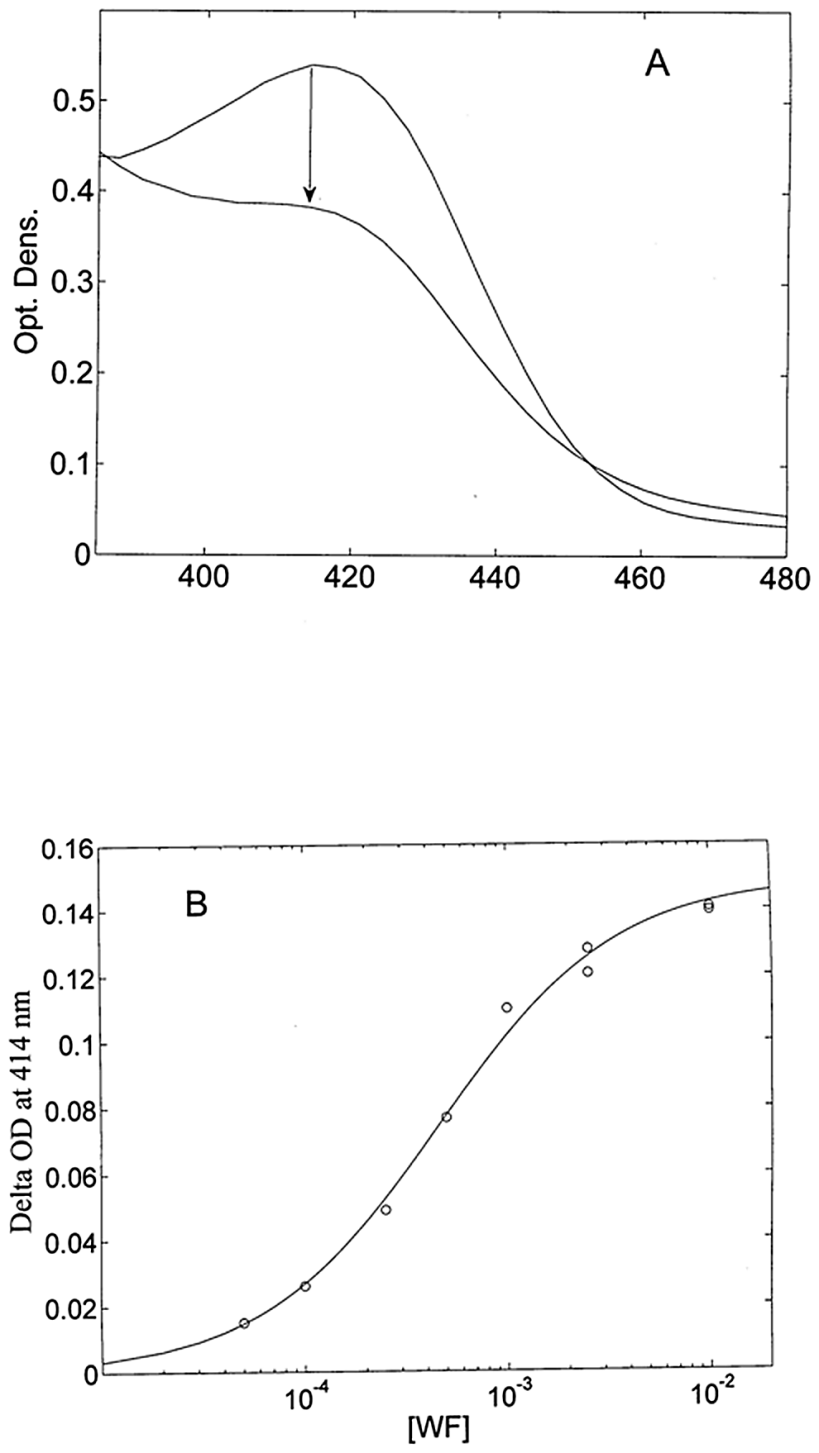
Thermodynamics of WF binding to HSA-heme-Fe(II). (A) Absorption spectral changes accompanying 1.0×10^−2^ M WF binding to HSA-heme-Fe(II), at pH 7.0 and 25°C. The equilibration time was 1 hr. The arrow indicates the direction of the absorption change. (B) Optical density changes at 414 nm for WF binding to HSA-heme-Fe(II), at pH 7.0 and 25°C. The continuous line was calculated by the non-linear least-squares fitting of data according to eq. (1) with K_WF_  =  2.2(±0.4)×10^3^ M^−1^. For details, see text.

HSA-heme-Fe(II) binds reversibly WF inducing the ∼ 20% decrease of the extinction coefficient of HSA-heme-Fe(II) at 414 nm and the shift of the peak wavelength from 414 nm to 411 nm (Fig. 4A), at pH 7.0 and 25°C. WF-dependent spectral changes are essentially completed at 1.0×10^−2^ M WF (Fig. 4B), indicating that this concentration saturates the WF binding site of HSA-heme-Fe(II). However, it must be underlined that the spectral change(s) following the addition of WF require(s) about 1 hr to reach the equilibrium condition, indicating the occurrence of a slow equilibration process (see above). Therefore, each experimental point reported in Fig. 4B refers to the optical density change(s) attained 1 hr after the addition of WF to HSA-heme-Fe(II).

WF binding to HSA-heme-Fe(II) follows a simple equilibrium, the Hill coefficient n being 1.01±0.02 (data not shown); therefore, data reported in Fig. 4B were analyzed in the framework of the minimum reaction mechanism depicted in Fig. 2. From the analysis of data according to eq. (1) the value of *K_WF_*  =  2.2(±0.4)×10^3^ M^−1^ was determined. Since we waited species re-equilibration after WF binding, we observed the overall process of WF binding to all three HSA-heme-Fe(II) populations [45]; therefore, the resulting equilibrium constant reflects the affinity of all three HSA-heme-Fe(II) species weighted by their relative percentage, and it guarantees that at 1×10^−2^ M WF concentration all HSA-heme-Fe(II) species are saturated with WF.

### Kinetics of WF binding to HSA-heme-Fe(II) and to HSA-heme-Fe(II)-CO

**Figure 5:**
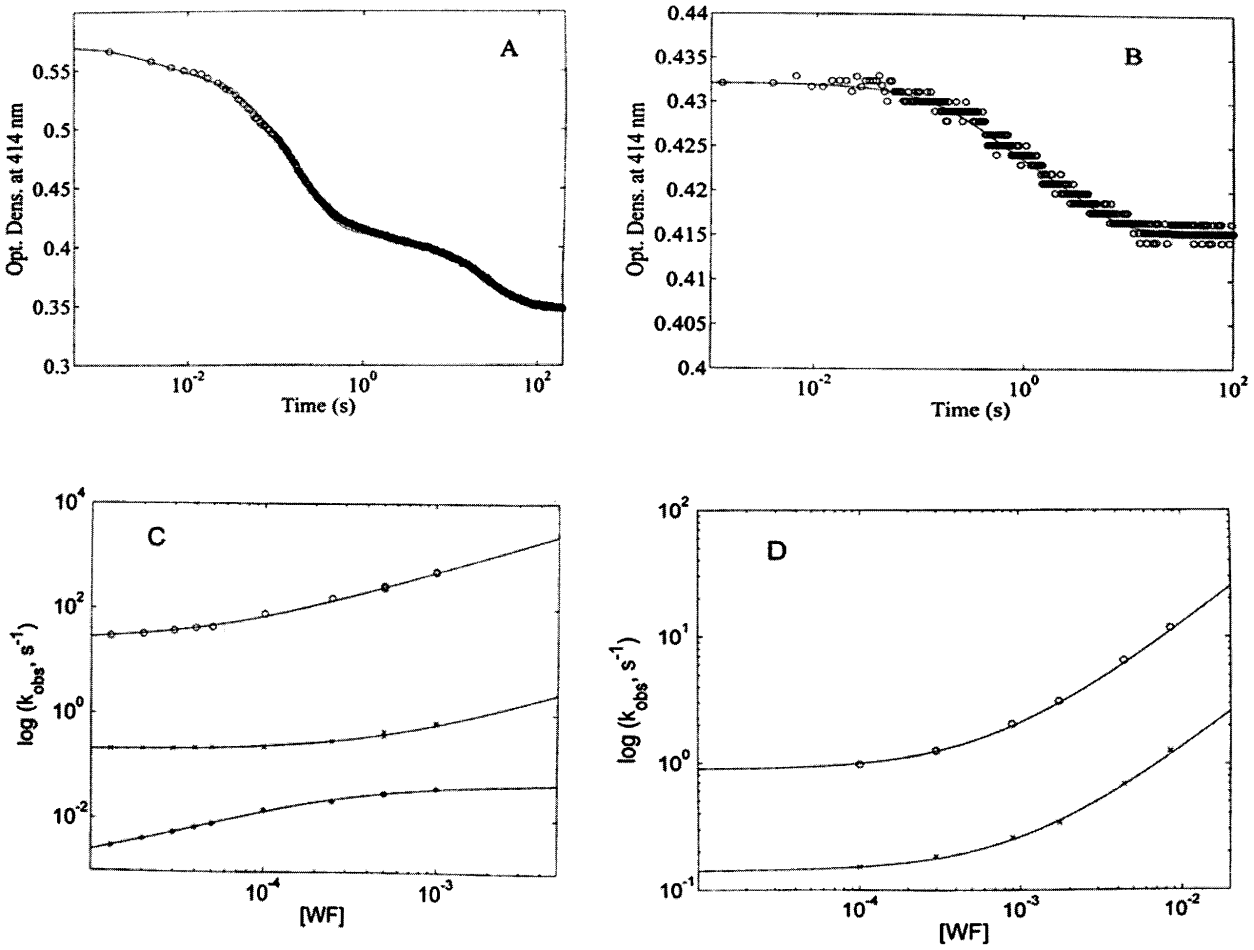
Kinetics of WF binding to HSA-heme-Fe(II) and HSA-heme-Fe(II)-CO. (A) Kinetic progress curve at 414 nm for WF ( =  2.5×10^−3^ M) binding to HSA-heme-Fe(II). Continuous line is the non-linear-least-squares fitting of data according to eq. (2), employing three exponentials (i.e., i  =  3). (B) Kinetic progress curve at 414 nm for WF ( =  4.0×10^−5 ^M) binding to HSA-heme-Fe(II)-CO. Continuous line is the non-linear-least-squares fitting of data according to eq. (2), employing two exponentials (i.e., i  =  2). (C) WF concentration dependence of observed rate constants for WF binding to 6cLS(1) (o), 5cHS(1) (x), and 4cIS (o). Continuous lines are the non-linear least-squares fitting of data according to eqs (3) (o,x) and (4b) (*). Kinetic parameters obtained from the analysis of data are reported in Table 1. (D) WF concentration dependence of the observed rate constants for WF binding to 6cLS(2) (o) and 6cLS(3) (x). Continuous lines are the non-linear least-squares fitting of data according to eq. (3). Kinetic parameters obtained from the analysis of data are reported in Table 1.

Figures 5A and 5B show kinetics of WF binding to HSA-heme-Fe(II) and HSA-heme-Fe(II)-CO, which have been analyzed according to eq. (2). However, it is worth recalling that WF does not bind to the heme-Fe(II) atom; therefore, the observed absorption changes reflect structural changes of the heme-Fe(II) group upon interaction of WF with Sudlow’s site I (i.e., the FA7 site, and possibly the FA2 cleft) [3], [54], [55].

Kinetics of WF binding to HSA-heme-Fe(II) is characterized by three exponentials (Fig. 5A) (as for CO binding, see Fig. S1A) [45], whose percentage closely mirrors what already reported for CO binding (therefore *n*  =  3 in eq. (2)). This behaviour indeed was expected, since the observed population distribution should be identical with that observed for CO association in the absence of WF [45]. On the other hand, kinetics of WF binding to HSA-heme-Fe(II)-CO displays only two exponentials (Fig. 5B) (as for CO dissociation from HSA-heme-Fe(II)-CO, see Fig. S1C) [45] whose percentage closely mirrors what observed for CO dissociation from HSA-heme-Fe(II)-CO in the absence of WF (therefore *n*  =  2 in eq. (2)). Altogether, the information on the number of reacting species and on their relative percentage reinforces the hypothesis that they indeed reflect the apparent equilibrium constants between the different conformations of HSA-heme-Fe(II) and HSA-heme-Fe(II)-CO.

WF binding to HSA-heme-Fe(II) and HSA-heme-Fe(II)-CO shows rate constants much slower than those for CO binding (Figs 5C and 5D and Table 1). The dependence of the rate constants over the explored WF concentration range allowed to determine the WF association and dissociation rate constants for the HSA-heme-Fe(II) and HSA-heme-Fe(II)-CO (see Figs. 5C and 5D).

**Table 1:** Kinetic parameters for CO and WF binding to different species of HSA-heme-Fe(II).

	*k_on_* (M^−1^ s^−1^)	*k_-L_* (s^−1^)	*k_on_/k_+L_*	*k_off_*
CO binding				
*^1^k_CO_* (4*cIS* + 5*cHS*(1))	4.0(±0.5)×10^6^	–	–	2.9(±0.4)×10^−2^
*^2^k_CO_* (6*cLS*(1))	–	27±3.9	6.1(±0.8)×10^5^	2.6(±0.4)×10^−1^
*^1^k_WFCO_* (WF-5*cHS*(1))	5.9(±0.7) ×10^5^	–	–	1.2(±0.3)×10^−1^
*^2^k_WFCO_* (WF-6*cLS*(1))	–	0.87±0.11	4.0(±0.5)×10^4^	1.6(±0.3)×10^−2^
WF binding				
*^1^k_WF_* (4*cIS* + 5*cHS*(1))	4.4(±0.6)×10^2^	–	–	2.0(±0.4)×10^−1^
*^2^k_WF_* (6*cLS*(1))	4.7(±0.7)×10^5^	–	–	2.4(±0.4)×10^1^
*^3^k_WF_*		0.049±0.006	3.5(±0.5)×10^3^	8.9(±1.3)×10^−4^
*^1^k_COWF_* (6*cLS*(2))	1.2(±0.3)×10^3^	–	–	8.9(±1.2)×10^−1^
*^2^k_COWF_* (6*cLS*(3))	1.2(±0.3)×10^2^	–	–	1.4(±0.3)×10^−1^

In particular, for WF binding to HSA-heme-Fe(II) we observed a very fast phase, amounting to about 10% of the total absorption change (see Fig. 5A), which should be referred to the same species characterized by the rate-limiting step behaviour reported for CO binding (see Fig. S1B). Therefore, the kinetic constant of WF binding to this species should be identified as *^2^k_obsWF_*; from the WF concentration dependence according to eq. (4), values of *^2^k_onWF_* ( =  (4.5±0.6)×10^5^ M^−1^ s^−1^) and *^2^k_offWF_* ( =  23.7±4.1 s^−1^) have been determined. A second slower phase, amounting to about 80% of the total absorption change (see Fig. 5A), displays as well a bimolecular behaviour (likely referring to the predominant species for CO binding, see Fig. S1A) and should be identified as *^1^k_obsWF_*. The dependence on WF concentration of the rate constant *^1^k_obsWF_* according to eq. (3) allowed to determine the *^1^k_onWF_* value ( =  (4.4±0.6)×10^2^ M^−1^ s^−1^). On the other hand, the third HSA-heme-Fe(II) species, amounting to about 10% of the total absorption change, shows a rate-limiting step in the reaction with WF (see Fig. 5C and Table 1), suggesting that WF binding to FA7 (and/or FA2) [3] is somehow limited by the displacement of some residue(s).

In the case of WF binding to HSA-heme-Fe(II)-CO, both species display a bimolecular behavior, thus the dependence of rate constants on WF concentration has been analysed according to eq. (3) (see Fig. 5D). From the relative percentage of the two phases, we can attribute the faster predominant rate constant, amounting to about 70% of the total absorption change (see Fig. 5B), to the species corresponding to *^1^k_offCO_* (see Fig. S1C). Therefore, we can identify this process with *^1^k_obsCOWF_*; from the dependence of *^1^k_obsCOWF_* on the WF concentration, values of *^1^k_onCOWF_* ( =  (1.2±0.3)×10^3^ M^−1^ s^−1^) and *^1^k_offCOWF_* ( =  0.89±0.12 s^−1^) were calculated according to eq. (3). The minority population, corresponding to about 30% of the total absorption change (see Fig. 5B), is instead characterized by *^2^k_onCOWF_*  =  (1.2±0.3)×10^2^ M^−1^ s^−1^ and *^2^k_offCOWF_*  =  0.14±0.03 s^−1^.

### Kinetics of CO binding to HSA-heme-Fe(II) and WF-HSA-heme-Fe(II)

Kinetics of CO binding to HSA-heme-Fe(II) displays at pH 7.0 a multiphasic reaction pattern, which can be accounted for by (at least) three exponentials (Fig. S1A); indeed, kinetics has been analyzed according to eq. (3) with *n*  =  3 [45] (see Material S1).

**Figure 6:**
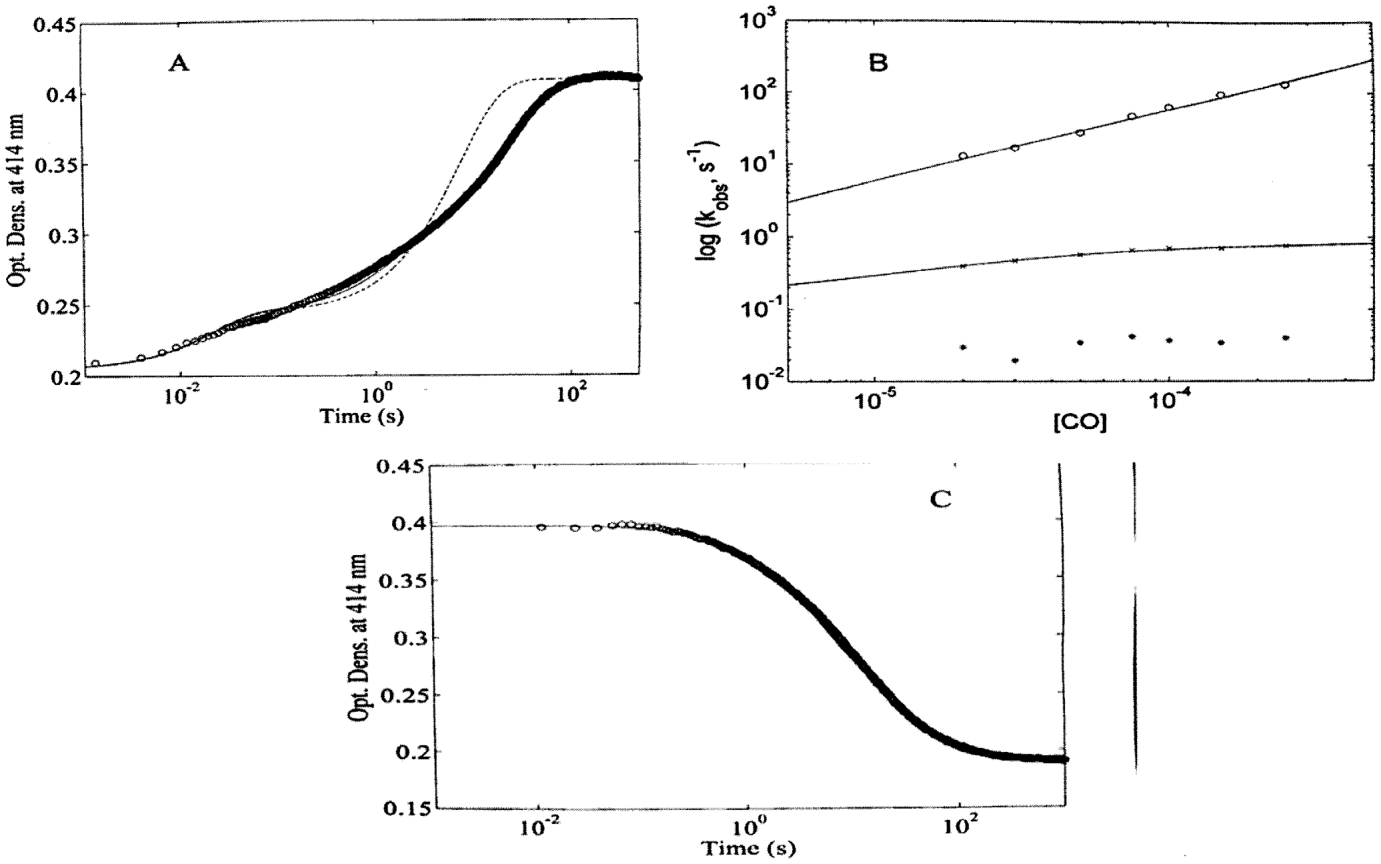
Kinetics of CO binding to WF-HSA-heme-Fe(II). (A) Kinetic progress curve at 414 nm for CO ( =  7.5×10^−5^ M) binding HSA-heme in the presence of WF ( =  1.0×10^−2^ M) equilibrated for 1 hr. Continuous line is the non-linear-least-squares fitting of data according to eq. (2), employing three exponentials (i.e., i  =  3). Dashed line is the non-linear-least-squares fitting of data according to eq. (2), employing two exponentials (i.e., i  =  2). (B) CO concentration dependence of the rate constants for CO binding in the presence of WF ( =  1.0×10^−2^ M) to WF-5cHS(1) (o) and WF-6cLS(1) (x); values described by “*” refer to the rate of species reequilibration (see text). Continuous lines are the non-linear least-squares fitting of data according to eqs (3) (o) and (4b) (x). Kinetic parameters obtained from the analysis are reported in Table 1. (C) Kinetic progress curve at 414 nm for CO dissociation from HSA-heme-Fe(II)-CO in the presence of WF ( =  1.0×10^−2^ M). The optical change corresponds to the displacement of CO by a large excess of NO. Continuous line represents the non-linear least-squares fitting of data according to eq. (2) (where k_obsCO_ is substituted by k_offCO_), employing two exponentials (i.e., i  =  2).

Kinetics of CO binding to WF-HSA-heme-Fe(II) displays a multiphasic reaction pattern, which can be accounted for by (at least) three exponentials (Fig. 6A) (therefore *n*  =  3 in eq. (2)). In contrast to CO binding to HSA-heme-Fe(II) [45], drastically different features occur in the carbonylation of WF-HSA-heme-Fe(II) (see Fig. S1A), over the whole CO concentration range explored. Indeed, in the absence of WF most of the HSA-heme-Fe(II) molecules (about 80%) are in the fast-reacting state (Fig. S1A) [45], whereas a marked reduction of the fast-reacting state occurs in the presence of 1.0×10^−2^ M WF (amounting to about 27%) (Fig. 6A). This change is accompanied by the increase of the percentage of the other two slow-reacting species, which become about 19% and 54%, respectively.

The WF-dependent variation of the percentage of the three HSA-heme-Fe(II) species (see Fig. 6A) indeed reflects the effect of the binding drug on the distribution of the three populations. Moreover, the slow spectral changes occurring upon WF addition to HSA-heme-Fe(II) (see above) are likely related to the slow re-distribution of the WF-HSA-heme-Fe(II) species.

The effect of CO concentration on values of the three pseudo-first-order rate constants for WF-HSA-heme-Fe(II) carbonylation in the presence of 1.0×10^−2^ M WF (i.e., *^1^k_obsWFCO_*, *^2^k_obsWFCO_*, and *^3^k_obsWFCO_*) (Fig. 6B) shows that, like in the absence of WF, the WF-HSA-heme-Fe(II) species undergoing the fastest carbonylation process (characterized by *^1^k_obsWFCO_* and corresponding to about 27% of the total absorption change, see Fig. 6A) follows a bimolecular behaviour. The analysis of data shown in Fig. 6B according to eq. (3) allowed to determine values of *^1^k_onWFCO_* ( =  (5.9±0.7)×10^5^ M^−1^ s^−1^) and *^1^k_offWFCO_* ( =  (1.2±0.3)×10^−1^ s^−1^) (see Table 1).

In contrast, the pseudo-first-order rate constant for the slowest process during the carbonylation of WF-HSA-heme-Fe(II) (characterized by *^3^k_obsWFCO_*  =  0.041±0.007 s^−1^) is independent of the CO concentration, as in the absence of WF (Figs. S1B and 6B, and Table 1).In the case of the intermediate reactive WF-HSA-heme-Fe(II) species (characterized by *^2^k_obsWFCO_*), both in the absence and presence of 1.0×10^−2^ M WF, values of the pseudo-first-order rate constant for WF-HSA-heme-Fe(II) carbonylation do not increase linearly with the CO concentration, but tend to level off as the CO concentration increases (Fig. 6B). This behaviour is similar to what observed in the absence of WF (see Fig. S1B) [45], suggesting that also in the presence of 1.0×10^−2^ M WF, one of the species displays a rate-limiting step, with a behaviour which can be described in Figure 3 and analyzed according to eq. (4a) [53]. The levelling off of the CO dependence of *^2^k_obsWFCO_* (as observed in Fig. 6B) allows to define accurately the value of *k_-L_*, but the distinct information on the relative values of *k_+L_’* and *^2^k_onWFCO_* cannot be obtained unless *^2^k_offWFCO_* is calculated independently.

CO dissociation kinetics from WF-HSA-heme-Fe(II)-CO is characterized by a biphasic kinetic pattern (Fig. 6C), suggesting that (like in the absence of WF, see Fig. S1C) [45] only two populations of WF-HSA-heme-Fe(II)-CO are sufficient to account for the observed behaviour.

As reported for CO dissociation kinetics in the absence of WF [45], values of the CO dissociation rate constants from the two WF-bound HSA-heme-Fe(II)-CO conformations display a 10-fold difference (i.e., *^1^k_offWFCO_*  =  0.12±0.03 s^−1^, and *^2^k_offWFCO_*  =  0.016±0.003 s^−1^), in the presence of 1.0×10^−2^ M WF. Therefore, binding of WF to HSA-heme-Fe(II)-CO species scarcely affects the CO dissociation process, since it brings about only a minor 2-fold variation for the CO dissociation process for either one of the two liganded conformations; this rules out a linkage between WF binding to FA7 (and/or FA2) [3] and the CO dissociation energy barrier. On the other hand, the two (WF-)HSA-heme-Fe(II)-CO conformations display a quite large difference of the energy barrier for the CO detachment, the 10-fold difference of rate constant amounting to an activation free energy change of about 4 kJ/mol.

### Spectroscopic characterization

**Figure 7:**
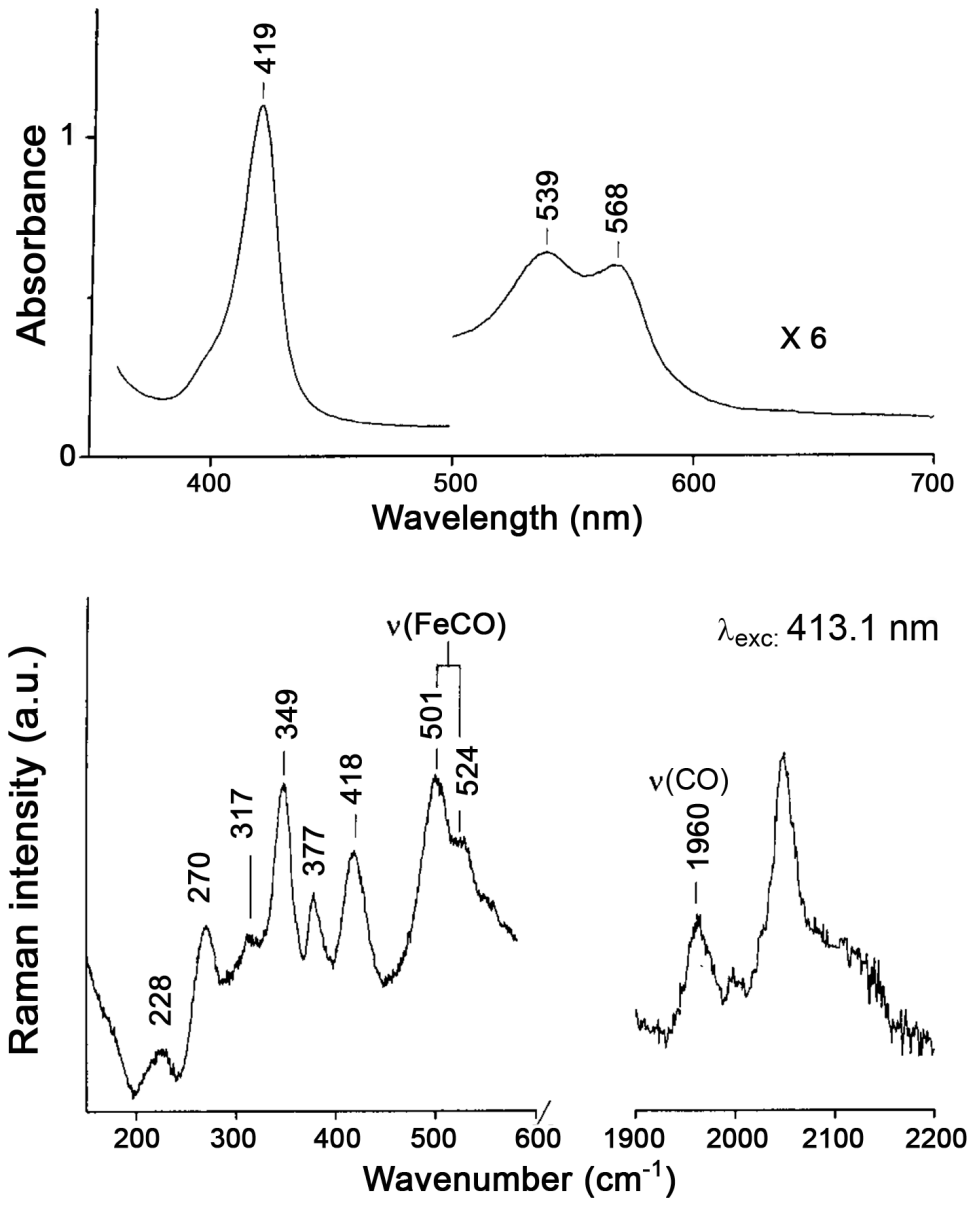
Spectroscopic properties of HSA-heme-Fe(II)-CO. Electronic absorption (Top) and RR spectra (bottom) of the CO complexes of HSA-heme-Fe(II)-CO at pH 7 in the presence of warfarin. RR spectra experimental conditions: 413.1 nm excitation; 1 and 3.3 cm^−1^ spectra resolution for the low and high frequency region, respectively; 3 mW laser power at the sample; average of 6 spectra (low frequency region) and 18 spectra (high frequency region) with 300 s integration time. The intensities are normalized to that of the ν_4_ band (not shown).

WF-HSA-heme-Fe(II)-CO gives rise to UV-Vis spectra characterized by bands at 419, 539, and 568 nm (Figure 7, top), similar to the spectra of HSA-heme-Fe(II)-CO at pH 10 [45] and of the ibuprofen-HSA-heme-Fe(II)-CO complex at neutral pH [23]. However, the UV-Vis spectrum of WF-HSA-heme-Fe(II)-CO is different from that of HSA-heme-Fe(II)-CO at pH 7.0. In the corresponding RR spectra (Figure 7, bottom) two [ν(Fe-CO)] stretching modes at 501 and 524 cm^−1^ are observed which shift to 494 and 520 cm^−1^, respectively in ^13^CO (data not shown). The corresponding [ν(CO)] stretching modes give rise to a broad band centred at 1960 cm^−1^ (1910 cm^−1^ in ^13^CO). As a matter of fact, CO is an excellent vibrational probe to investigate the heme cavity because Fe-CO back-bonding (from the Fe dπ electrons to the empty CO π* orbitals) is modulated by polar interactions with protein residues and by variations in the donor strength of the trans axial ligand. The electrostatic field generated by the polar distal pocket alters the electron distribution in the Fe-CO unit changing the bond order of the C-O bond, which can be monitored by the CO and Fe-C stretching frequencies. The ν(Fe-C) and ν(CO) stretching frequencies are inversely correlated and, from the ν(Fe-C) / ν(CO) position along the correlation line, information on the type and the strength of distal polar interactions are obtained. Moreover, changes in the trans ligand donor strength shift the correlation line to higher or lower position.

**Figure 8:**
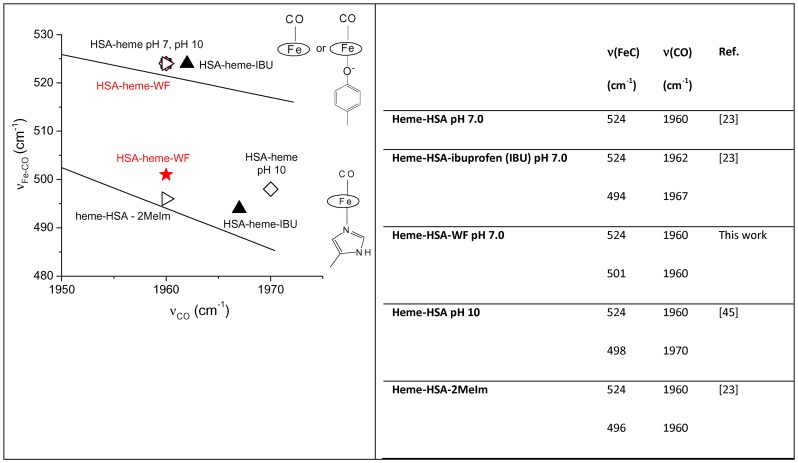
Plot of the ν(FeC) versus ν(CO) frequencies of HSA-heme-Fe(II) under various experimental conditions . Left: Plot of the ν(FeC) versus ν(CO) frequencies observed in the CO complexes of heme-HSA under various experimental conditions (right). The lower line indicates the back-bonding correlation line for six-coordinate CO heme proteins with imidazole as sixth ligand, as given in ref. [49]. The upper line represents five-coordinate, with no trans ligand, or six-coordinate CO heme proteins with weak trans ligands as Tyr [23].

Figure 8 shows the plot of the ν(Fe-CO) and ν(CO) frequencies observed in the HSA-heme-Fe(II)-CO complexes under various experimental conditions. The lines were obtained according to literature (see eq. 1 reported in ref. [56]). The slope represents the back-bonding sensitivity of ν(Fe-C). The ν(Fe-CO) stretching mode at 524 cm^−1^ and the ν(CO) stretch at 1960 cm^−1^ are located above the His line of the ν(Fe–CO)/ ν (CO) back-bonding correlation, since the proximal ligand is either weak [57] or absent [58]. The stretching mode at 501 cm^−1^ and the ν(CO) stretch at 1960cm^−1^ fall on the His ν(Fe–CO)/ν (CO) back-bonding correlation line, close to values of HSA-heme-Fe(II)-CO at alkaline pH and at neutral pH in the presence of 2-methylimidazole or ibuprofen, in which the trans axial ligand is an imidazole or His [23], [45]. These data confirm that in the presence of WF at neutral pH a major conformational change occurs in the heme pocket of HSA-heme-Fe(II)-CO, allowing the His146 residue to coordinate the heme-Fe(II) atom via its nitrogen atom. The formation of the Fe-N bond has been confirmed by the presence of the ν(Fe-Im) stretching mode upon photolysis of CO in the laser beam (data not shown). In fact, in the low frequency region of RR spectra obtained with 441.6 nm excitation of the WF-HSA-heme-Fe(II)-CO complex at high laser power (35mW), CO is partially photolyzed giving rise to WF-HSA-heme-Fe(II) which is characterized by a strong band ν(Fe-Im) mode at 218 cm^−1^.

## Discussion

HSA could be considered as a paradigm for monomeric allosteric proteins, since the conformational adaptability of HSA involves more than the immediate vicinity of the binding site(s) [1], [3], [11], [17], [20], [23], [28], [30], [31], [33], [35], [40], [43], [44], [54], [55], [59]-[65]. In this work, we have characterized quantitatively the reciprocal allosteric modulation between the WF and CO binding to HSA-heme-Fe(II). The choice of these two ligands (i.e., CO and WF) is related to (i) the relevant physiological role of HSA in heme scavenging under physiological and pathological condition [3], (ii) the peculiar role of CO as a neurotransmitter, being involved for example in the circadian cycle [66], [67], and (iii) the widespread utilization of warfarin as an anticoagulant drug [68].

Observations here reported clearly indicate that HSA-heme-Fe(II) is characterized by multiple conformational states and/or heme coordination forms, which display different CO and WF binding features, underlying a complex functional allosteric modulation.

The occurrence of three exponentials for the quantitative description of the CO association kinetic progress curves and of two exponentials for the CO dissociation time courses has been recently interpreted [45] as referable to a complex network of ligand- and proton-linked interactions between the heme and the neighbouring residues of the FA1 site (i.e., where the heme binds). In unliganded HSA-heme-Fe(II), three species occur, namely (i) a four-coordinated form (i.e., 4*cIS*), (ii) a five-coordinated form with Tyr161 as axial ligand (i.e., 5*cHS*(1)) and (iii) a six-coordinated form with Tyr161 and His146 as axial ligands (i.e., 6*cLS*(1)) [23]. In addition, three putative CO-bound species (originating from CO binding to the three unliganded species) occur, namely (i) a five-coordinated form upon CO binding to 4*cIS* (i.e., 5*cHS*(2)), (ii) a six-coordinated form upon CO binding to 5*cHS*(1) (i.e., 6*cLS*(2)) and (iii) a six-coordinated form with His146 as a sixth axial ligand (i.e., 6*cLS*(3)), which likely originates from CO binding to the form 6*cLS*(1) on displacement of Tyr161, ending up with a CO-bound form where the proximal ligand is His146. This behaviour, which is also supported by resonance Raman spectra (see Figs. 7, 8, S1A and S1B), can be accounted only assuming that [45]: (i) in unliganded HSA-heme-Fe(II) at pH 7.0 the predominant species is 5*cHS*(1), which is in fast equilibrium with the species 4*cIS* but in slow equilibrium with the 6*cLS*(1) species; (ii) at pH 7.0, the CO-bound 5*cHS*(2) species is very unstable and first quickly converts completely to the 6*cLS*(2) species, which also shifts slowly toward the 6*cLS*(3) species, giving rise to the very slow CO-independent process (see Fig. S1C); and (iii) at pH 7.0, about 70% of the HSA-heme-Fe(II)-CO molecules is represented by the 6*cLS*(2) species.

**Figure 9:**
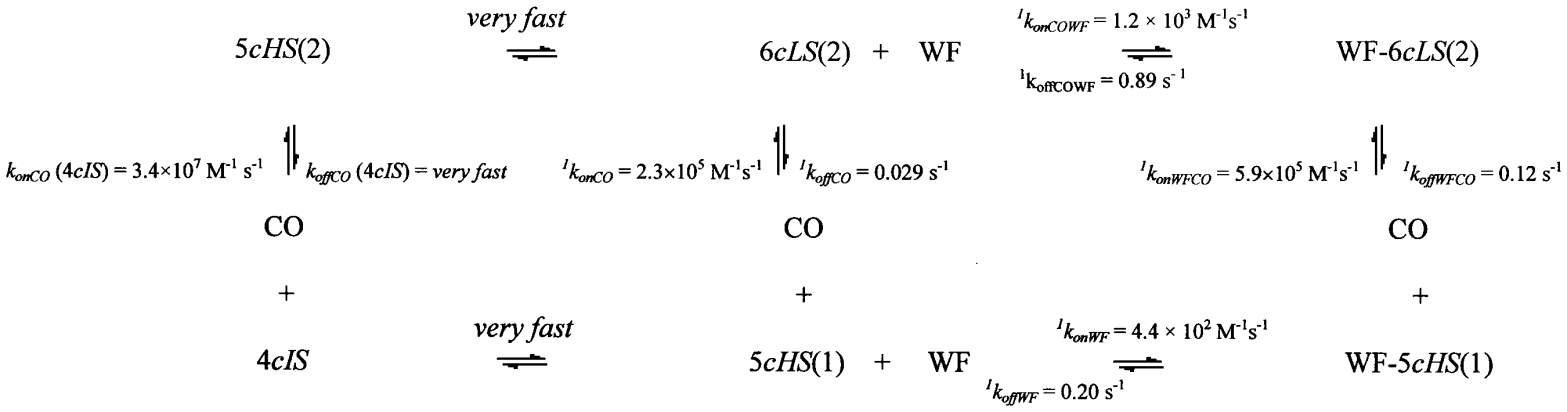
Thermodynamic and kinetic scheme correlating CO binding to 4*cIS*, 5*cHS*(1) and WF-5*cHS*(1) with WF binding to 5*cHS*(1) and 6*cLS*(2).

**Figure 10:**
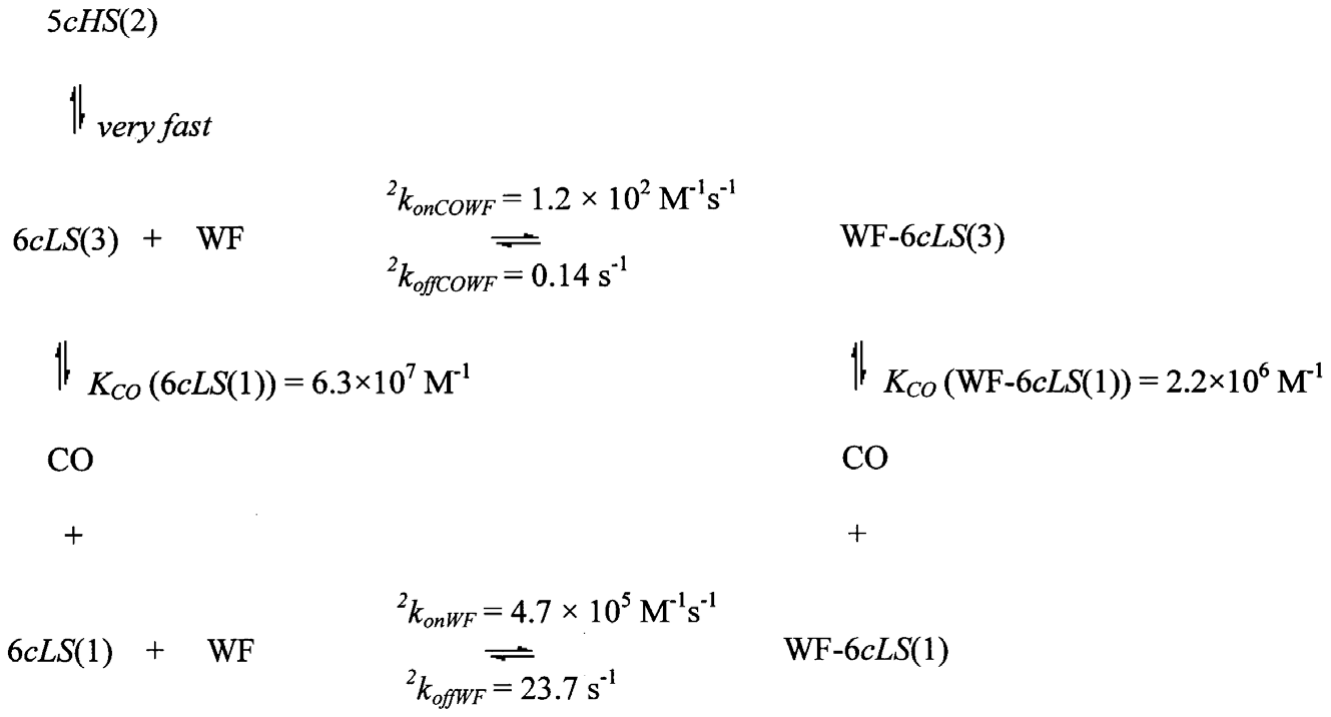
Thermodynamic and kinetic scheme correlating CO binding to 6*cLS*(1) and WF-6*cLS*(1) with WF binding to 6*cLS*(1) and 6*cLS*(3).

These informations have been now implemented with those of CO binding to WF-HSA-heme-Fe(II) and of WF binding to both HSA-heme-Fe(II) and HSA-heme-Fe(II)-CO; the resulting thermodynamic schemes for the two stable species 5*cHS*(1) (and its CO-bound form 6*cLS*(2), see Figure 9) and 6*cLS*(1) (and its CO-bound form 6*cLS*(3), see Figure 10) allow several considerations.

On the basis of thermodynamic considerations (Fig. 9), the CO equilibrium affinity for 5*cHS*(1) (*K_CO_* (5*cHS*(1))  =  8.0(±1.1)×10^6^ M^−1^) turns out to be slightly higher than for WF-5*cHS*(1) (*K_CO_* (WF-5*cHS*(1))  =  4.9(±0.7)×10^6^ M^−1^), envisaging a *k_onCO_* (5*cHS*(1))  =  2.3(±0.4)×10^5^ M^−1^ s^−1^. This value is considerably slower than that actually observed (*k_onCO_*  =  4.0(±0.5)×10^6^ M^−1^ s^−1^, see Table 1); however, this discrepancy is due to the fast equilibrium between the 4*cIS* and 5*cHS*(1) species, such that the resulting association rate constant
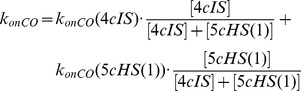
(5)


Therefore, if we substitute in eq. (5) to *k_onCO_* (5*cHS*(1)) the value of 2.3×10^5^, as obtained from Fig. 9, and to *k_onCO_* (4*cIS*) the value of 3.4×10^7^, as reported for a four-coordinate heme inside a protein [69], the experimentally observed value of *k_onCO_* is 4.0(±0.5)×10^6^ M^−1^ s^−1^, employing the 1:8 ratio between populations of 4*cIS* and 5*cHS*(1) in HSA-heme-Fe(II).

Furthermore, from Figure 9 it comes out that CO binding to both 5*cHS*(1) and WF-5*cHS*(1) shows association rate constants closely similar to what observed for mammalian myoglobins (Mbs) [52]. On the other hand, the CO dissociation rate from WF-6*cLS*(2) is significantly faster than in mammalian Mbs [52], possibly reflecting a weaker heme-protein proximal bond [70], whereas in the absence of WF the CO dissociation rate from 6*cLS*(2) is closely similar to those reported for mammalian Mbs [52]. This suggests that WF binding has a significant effect on CO dissociation, decreasing by about 3.5 kJ/mol the energy barrier for the CO detachment from the heme-Fe atom.

As shown in Fig. 9, WF binding to 5*cHS*(1) is somewhat slower than drug binding to 6*cLS*(2), even though 5*cHS*(1) displays a slightly higher affinity for WF (*K_WF_* (5*cHS*(1))  =  2.2(±0.4)×10^3^ M^−1^) with respect to 6*cLS*(2) (*K_WF_* (6*cLS*(2))  =  1.35(±0.31)×10^3^ M^−1^). Therefore, equilibrium CO binding has a moderate allosteric effect for the interaction of WF with 5*cHS*(1), in spite of the significant effect on CO dissociation upon WF binding (see above).

A completely different behaviour has been observed for the 6*cLS*(1) species (Figure 10), such that the WF binding rate constant to 6*cLS*(1) is about 4000-fold faster than for 6*cLS*(3) ligation, with a WF binding equilibrium affinity for 6*cLS*(1) (*K_WF_* (6*cLS*(1))  =  2.0(±0.3)×10^4^ M^−1^) which is 30-fold higher than for 6*cLS*(3) (*K_WF_* (6*cLS*(3))  =  7.1(±0.9)×10^2^ M^−1^), indeed suggesting a strong CO-linked allosteric effect for the six-coordinated 6*cLS*(1) species.

However, it must be underlined that the interaction of WF with 6*cLS*(1) is also much faster (by about 1000-fold) with respect to all other species, including the five-coordinated 5*cHS*(1) form (see Fig. 9 and Table 1). Thus, it comes out that six-coordination of the heme-Fe atom at the FA1 site lowers the free energy kinetic barrier for WF association by about 17.2 kJ/mol, with a much more negative binding free energy for 6*cLS*(1) (*ΔG_WF_*  =  −24.4 kJ/mol) than for the 5*cHS*(1) species (*ΔG_WF_*  =  −19.0 kJ/mol). The overall process appears to be shifted in favour of the WF-6*cLS*(1) species also because upon WF binding essentially all the 4*cIS* species disappears in favour of the WF-6*cLS*(1) form.

The functional difference, and thus the allosteric modulation linked to the proximal ligand of the heme-Fe atom, comes out clear on comparing the species where the proximal axial ligand is either Tyr161 (Fig. 9) or His146 (Fig. 10). Therefore, in the case of WF binding to HSA-heme-Fe(II)-CO the CO-bound 6*cLS*(2) species appears to react more quickly with WF (showing a *^1^k_onCOWF_*  =  (1.2±0.3)×10^3^ M^−1^ s^−1^ and *^1^k_offCOWF_*  =  0.89±0.12 s^−1^) than the CO-bound 6*cLS*(3) (with a *^2^k_onCOWF_*  =  (1.2±0.3)×10^2^ M^−1^ s^−1^ and *^2^k_offCOWF_*  =  0.14±0.03 s^−1^). This indicates that the energy barrier for the access of WF to the FA7 (and/or FA2) [3] site(s) is much lower when the heme is coordinated to Tyr161 than to His146, being referable to a decrease of the energy barrier of about 5.6 kJ/mol. On the other hand, the WF binding equilibrium constant displays a moderate difference between the two species, suggesting that WF binding displaces only slightly the equilibrium in favour of the WF-6*cLS*(2) form.

The effect of the proximal heme-Fe ligand can be observed also in the case of HSA-heme-Fe(II)-CO dissociation, since the biphasic kinetic progress curve (see Fig. S1C) can be accounted for by the occurrence of two CO-bound species, namely 6*cLS*(2) and 6*cLS*(3) (also detected by resonance Raman spectroscopy, see Fig. 8), whose rate constants differ in the absence of WF by one order of magnitude (i.e., *k_offCO_*  =  0.029±0.004 s^−1^ and 0.26±0.04 s^−1^) (see Table 1). Therefore, the energy barrier for the CO detachment is quite marked between the two forms, the 10-fold difference of *k_offCO_* amounting to an activation free energy lower by about 4 kJ/mol when His 146 is the proximal heme-Fe ligand. The situation is completely changed upon addition of WF, since the rate of CO dissociation from WF-6*cLS*(2) (*^1^k_offWFCO_*  =  0.12±0.03 s^−1^, see Table 1) is about ten-fold faster than that for decarbonylation WF-*6cLS*(3) (*^2^k_offWFCO_*  =  0.016±0.003 s^−1^, see Table 1), clearly indicating that WF binding stabilizes the bound CO when His146 is the proximal ligand with an enhancement of 5 kJ/mol for the energy barrier of CO dissociation.

The interrelationship between WF binding and the strength of the heme-Fe atom proximal bond emerges also when CO binding is rate-limited by the displacement of the endogenous ligand (likely Tyr161). Thus, in the presence of WF the rate of displacement of the endogenous ligand is about 30-fold slower (see Figure 6B and Table 1), clearly indicating that the binding of WF to FA7 (and/or FA2) [3] enhances by about 8.3 kJ/mol the energy barrier for dissociation of the heme-Fe atom axial ligand Tyr161 (to allow CO binding and the formation of the WF-6cLS(3) species with His146 as the axial ligand).

Both CO binding processes to HSA-heme-Fe(II) [45] and to WF-HSA-heme-Fe(II) are also characterized by a third very slow phase (characterized by a relaxation rate *h*  =  0.044±0.007 s^−1^, in the absence of WF, and 0.039±0.006 s^−1^, in the presence of 1.0×10^−2^ M WF, and a much larger absorption change amplitude at 414 nm in the case of WF-HSA-heme-Fe(II) carbonylation, see Fig. 6A and S1B), which should not reflect a population in the unliganded form, referring instead to the slow re-equilibration process between the CO-bound 6*cLS*(2) and the 6*cLS*(3) species (or between the CO-bound WF-6*cLS*(2) and the WF-6*cLS*(3) specie). The much larger apparent slower phase observed during CO binding to WF-HSA-heme-Fe(II) indeed might be related to the extensive rearrangement of the CO-bound species between the WF-6*cLS*(2) and the WF-6*cLS*(3) forms, a feature likely related to the fact that the 4*cIS* species is fully destabilized by WF binding, bringing about a shift in favour of the WF-6*cLS*(1) species.

As a whole, the allosteric effect exerted by WF binding to its site(s) seems to be much more pronounced in the unliganded form of HSA-heme-Fe(II), inducing a strong stabilization of the hexa-coordinated form mostly by strengthening the Fe-Tyr161 axial bond, which in turn renders more difficult the detachment of the axial Tyr161-heme coordination bond upon CO binding. On the other hand, CO binding abolishes the endogenous hexa-coordination of the heme-Fe atom, enhancing the kinetic free energy barrier for WF association; this effect is particularly marked for the 6cLS(2) species, suggesting that the heme-Fe atom coordination by Tyr161 lowers by about 20 kJ/mol the energy barrier for WF binding. Therefore, the allosteric mechanism operating in HSA-heme-Fe(II) between the FA1 and WF binging site(s) appears modulated mostly by the axial ligands of the heme, with Tyr161 being the most favourable axial ligand for WF interaction.

## Supporting Information

Figure S1 (*panel A*) Kinetic progress curve of CO binding ([CO]  =  4.0×10^-5^ ) to 5.0×10^-6^ M HSA-heme-Fe(II) at pH 7.0 and 25°C in 1.0×10^-1^ M phosphate buffer. Continuous line: non-linear least-squares fitting of experimental data according to eq. (2) employing *i*  =  3. Dashed line: non-linear least-squares fitting of experimental data according to eq. (2) employing *i*  =  2. (*panel B*) CO concentration dependence of the rate constants for CO binding to HSA-heme-Fe(II). Continuous lines are the non-linear least-squares fitting of data according to eqs (4) (o) and (5b) (x). Kinetic parameters obtained from the analysis of data are reported in Table 1. (*panel C*) Kinetic progress curve at 414 nm for CO dissociation from HSA-heme-Fe(II)-CO. The optical change corresponds to the displacement of the bound CO by a large excess of NO (see Experimental Procedure). Continuous line represents the non-linear least-squares fitting of data according to eq. (2) (where *k_obsCO_* is substituted by *k_offCO_*), employing two exponentials (*i.e.*, *i*  =  2).
(TIF)

Material S1 **Supplementary materials.**
(DOC)

## References

[1] Peters T Jr (1996) All about Albumin: Biochemistry, Genetics and Medical Applications. Academic Press, San Diego and London.

[2] Minchiotti L, Galliano M, Kragh-Hansen U, Peters T Jr (2008) Mutations and polymorphisms of the gene of the major human blood protein, serum albumin. Hum Mutat 29: 1007–1016. 18459107 10.1002/humu.20754

[3] Fanali G, di Masi A, Trezza V, Marino M, Fasano M, et al (2012) Human serum albumin: from bench to bedside. Mol Aspects Med 33: 209–290. 22230555 10.1016/j.mam.2011.12.002

[4] He X, Carter DC (1992) Atomic structure and chemistry of human serum albumin. Nature 358: 209–215. 1630489 10.1038/358209a0

[5] Carter DC, Ho JX (1994) Structure of serum albumin. Adv Protein Chem 45: 153–203. 8154369 10.1016/s0065-3233(08)60640-3

[6] Curry S, Mandelkov H, Brick P, Franks N (1998) Crystal structure of human serum albumin complexed with fatty acid reveals an asymmetric distribution of binding sites. Nat Struct Biol 5: 827–835. 9731778 10.1038/1869

[7] Sugio S, Kashima A, Mochizuki S, Noda M, Kobayashi K (1999) Crystal structure of human serum albumin at 2.5 Å resolution. Protein Eng 12: 439–446. 10388840 10.1093/protein/12.6.439

[8] Bhattacharya AA, Curry S, Franks NP (2000) Binding of the general anesthetics propofol and halothane to human serum albumin. High resolution crystal structures. J Biol Chem 275: 38731–38738. 10940303 10.1074/jbc.M005460200

[9] Bhattacharya AA, Grüne T, Curry S (2000) Crystallographic analysis reveals common modes of binding of medium and long-chain fatty acids to human serum albumin. J Mol Biol 303: 721–732. 11061971 10.1006/jmbi.2000.4158

[10] Petitpas I, Bhattacharya AA, Twine S, East M, Curry S (2001) Crystal structure analysis of warfarin binding to human serum albumin: anatomy of drug site I. J Biol Chem 276: 22804–22809. 11285262 10.1074/jbc.M100575200

[11] Curry S (2002) Beyond expansion: structural studies on the transport roles of human serum albumin, Vox Sang. 83 (Suppl. 1)315–319. 10.1111/j.1423-0410.2002.tb05326.x 12617161

[12] Wardell M, Wang Z, Ho JX, Robert J, Rüker F, et al (2002) The atomic structure of human methemalbumin at 1.9 Å. . Biochem Biophys Res Commun 291: 813–819. 11866438 10.1006/bbrc.2002.6540

[13] Petitpas I, Petersen CE, Ha CE, Bhattacharya AA, Zunszain PA, et al (2003) Structural basis of albumin-thyroxine interactions and familial dysalbuminemic hyperthyroxinemia. Proc Natl Acad Sci USA 100: 6440–6445. 12743361 10.1073/pnas.1137188100 PMC164465

[14] Zunszain PA, Ghuman J, Komatsu T, Tsuchida E, Curry S (2003) Crystal structural analysis of human serum albumin complexed with hemin and fatty acid. BMC Struct Biol 3: 6. 12846933 10.1186/1472-6807-3-6 PMC166163

[15] Sakurai Y, Ma SF, Watanabe H, Yamaotsu N, Hirono S, et al (2004) Esterase-like activity of serum albumin: characterization of its structural chemistry using p-nitrophenyl esters as substrates. Pharm Res 21: 285–292. 15032310 10.1023/b:pham.0000016241.84630.06

[16] Fasano M, Curry S, Terreno E, Galliano M, Fanali G, et al (2005) The extraordinary ligand binding properties of human serum albumin. IUBMB Life 57: 787–796. 16393781 10.1080/15216540500404093

[17] Ascenzi P, Bocedi A, Notari S, Fanali G, Fesce R, et al (2006) Allosteric modulation of drug binding to human serum albumin. Mini Rev Med Chem 6: 483–489. 16613585 10.2174/138955706776361448

[18] Zunszain PA, Ghuman J, McDonagh AF, Curry S (2008) Crystallographic analysis of human serum albumin complexed with 4Z,15E-bilirubin-IXalpha. J Mol Biol 381: 394–406. 18602119 10.1016/j.jmb.2008.06.016 PMC2568863

[19] Simard JR, Zunszain PA, Hamilton JA, Curry S (2006) Location of high and low affinity fatty acid binding sites on human serum albumin revealed by NMR drug-competition analysis. J Mol Biol 361: 336–351. 16844140 10.1016/j.jmb.2006.06.028

[20] Bocedi A, Notari S, Menegatti E, Fanali G, Fasano M, et al (2005) Allosteric modulation of anti-HIV drug and ferric heme binding to human serum albumin. FEBS J 272: 6287–6296. 16336266 10.1111/j.1742-4658.2005.05015.x

[21] Fasano M, Fanali G, Leboffe L, Ascenzi P (2007) Heme binding to albuminoid proteins is the result of recent evolution. IUBMB Life 59: 436–440. 17654119 10.1080/15216540701474523

[22] Fasano M, Baroni S, Vannini A, Ascenzi P, Aime S (2001) Relaxometric characterization of human hemalbumin. J Biol Inorg Chem 6: 650–658. 11472028 10.1007/s007750100242

[23] Nicoletti FP, Howes BD, Fittipaldi M, Fanali G, Fasano M, et al (2008) Ibuprofen induces an allosteric conformational transition in the heme complex of human serum albumin with significant effects on heme ligation. J Am Chem Soc 130: 11677–11688. 18681435 10.1021/ja800966t

[24] Sudlow G, Birkett DJ, Wade DN (1975) The characterization of two specific drug binding sites on human serum albumin. Mol Pharmacol 11: 824–832. 1207674

[25] Sudlow G, Birkett DJ, Wade DN (1976) Further characterization of specific drug binding sites on human serum albumin. Mol Pharmacol 12: 1052–1061. 1004490

[26] Diana FJ, Veronich K, Kapoor AL (1989) Binding of nonsteroidal anti-inflammatory agents and their effect on binding of racemic warfarin and its enantiomers to human serum albumin. J Pharm Sci 78: 195–199. 2724076 10.1002/jps.2600780304

[27] Yamasaki K, Maruyama T, Yoshimoto K, Tsutsumi Y, Narazaki R, et al (1999) Interactive binding to the two principal ligand binding sites of human serum albumin: effect of the neutral-to-base transition. Biochim Biophys Acta 1432: 313–323. 10407153 10.1016/s0167-4838(99)00098-9

[28] Baroni S, Mattu M, Vannini A, Cipollone R, Aime S, et al (2001) Effect of ibuprofen and warfarin on the allosteric properties of haem-human serum albumin: a spectroscopic study. Eur J Biochem 268: 6214–6220. 11733017 10.1046/j.0014-2956.2001.02569.x

[29] Ghuman J, Zunszain PA, Petitpas I, Bhattacharya AA, Otagiri M, et al (2005) Structural basis of the drug-binding specificity of human serum albumin. J Mol Biol 353: 38–52. 16169013 10.1016/j.jmb.2005.07.075

[30] Chuang VTG, Otagiri M (2002) How do fatty acids cause allosteric binding of drugs to human serum albumin? Pharm Res 19: 1458–1464. 12425462 10.1023/a:1020496314081

[31] Fanali G, Fesce R, Agrati C, Ascenzi P, Fasano M (2005) Allosteric modulation of myristate and Mn(III)heme binding to human serum albumin: optical and NMR spectroscopy characterization. FEBS J 272: 4672–4683. 16156788 10.1111/j.1742-4658.2005.04883.x

[32] Kragh-Hansen U, Watanabe H, Nakajou K, Iwao Y, Otagiri M (2006) Chain length-dependent binding of fatty acid anions to human serum albumin studied by site-directed mutagenesis. J Mol Biol 363: 702–712. 16979183 10.1016/j.jmb.2006.08.056

[33] Fanali G, Bocedi A, Ascenzi P, Fasano M (2007) Modulation of heme and myristate binding to human serum albumin by anti-HIV drugs. An optical and NMR spectroscopic study. FEBS J 274: 4491–4502. 17725715 10.1111/j.1742-4658.2007.05978.x

[34] Fasano M, Fanali G, Fesce R, Ascenzi P (2008) Human serum haem-albumin: an allosteric ‘chronosteric’ protein. In: Bolognesi M, di Prisco G, Verde C (eds) Dioxygen binding and sensing proteins. Springer, Heidelberg, pp. 121–131.

[35] Fanali G, Pariani G, Ascenzi P, Fasano M (2009) Allosteric and binding properties of Asp1-Glu382 truncated recombinant human serum albumin - An optical and NMR spectroscopic investigation. FEBS J 276: 2241–2250. 19298387 10.1111/j.1742-4658.2009.06952.x

[36] Wilting J, van der Giesen WF, Janssen LH, Weideman MM, Otagiri M, et al (1980) The effect of albumin conformation on the binding of warfarin to human serum albumin. The dependence of the binding of warfarin to human serum albumin on the hydrogen, calcium, and chloride ion concentrations as studied by circular dichroism, fluorescence, and equilibrium dialysis. J Biol Chem 255: 3032–3037. 7358725

[37] Janssen LH, Van Wilgenburg MT, Wilting J (1981) Human serum albumin as an allosteric two-state protein: evidence from effects of calcium and warfarin on proton binding behaviour. Biochim Biophys Acta 669: 244–250. 7284438 10.1016/0005-2795(81)90247-6

[38] Dockal M, Chang M, Carter DC, Rüker F (2000) Five recombinant fragments of human serum albumin. Tools for the characterization of the warfarin binding site. Protein Sci 9: 1455–1465. 10975567 10.1110/ps.9.8.1455 PMC2144726

[39] Monzani E, Bonafé B, Fallarini A, Redaelli C, Casella L, et al (2001) Enzymatic properties of hemalbumin. Biochim Biophys Acta 1547: 302–312. 11410286 10.1016/s0167-4838(01)00192-3

[40] Ascenzi P, Bocedi A, Bolli A, Fasano M, Notari S, et al (2005) Allosteric modulation of monomeric proteins. Biochem Mol Biol Educ 33: 169–176. 21638571 10.1002/bmb.2005.494033032470

[41] Komatsu T, Ohmichi N, Nakagawa A, Zunszain PA, Curry S, et al (2005) O_2_ and CO binding properties of artificial hemoproteins formed by complexing iron protoporphyrin IX with human serum albumin mutants. J Am Chem Soc 127: 15933–15942. 16277537 10.1021/ja054819u

[42] Ascenzi P, Fasano M (2007) Abacavir modulates peroxynitrite-mediated oxidation of ferrous nitrosylated human serum heme-albumin. Biochem Biophys Res Commun 353: 469–474. 17188651 10.1016/j.bbrc.2006.12.041

[43] Ascenzi P, Imperi F, Coletta M, Fasano M (2008) Abacavir and warfarin modulate allosterically kinetics of NO dissociation from ferrous nitrosylated human serum heme-albumin. Biochem Biophys Res Commun 369: 686–691. 18307975 10.1016/j.bbrc.2008.02.077

[44] Ascenzi P, di Masi A, De Sanctis G, Coletta M, Fasano M (2009) Ibuprofen modulates allosterically NO dissociation from ferrous nitrosylated human serum heme-albumin by binding to three sites. Biochem Biophys Res Commun 387: 83–86. 19559669 10.1016/j.bbrc.2009.06.117

[45] Cao Y, Nicoletti FP, De Sanctis G, Bocedi A, Ciaccio C, et al (2012) Evidence for pH-dependent multiple conformers in iron(II) heme-human serum albumin: spectroscopic and kinetic investigation of carbon monoxide binding. J Biol Inorg Chem 17: 133–147. 21894504 10.1007/s00775-011-0837-0

[46] Chen RF (1967) Removal of fatty acids from serum albumin by charcoal treatment. J Biol Chem 242: 173–181. 6016603

[47] Sogami M, Foster JF (1968) Isomerization reactions of charcoal-defatted bovine plasma albumin. The N-F transition and acid expansion. Biochemistry 7: 2172–2182. 5690710 10.1021/bi00846a020

[48] Cabrera-Crespo J, Goncalves VM, Martins EA, Grellet S, Lopes AP, et al (2000) Albumin purification from human placenta. Biotechnol Appl Biochem 31: 101–106. 10744954 10.1042/ba19990095

[49] Bradford M (1976) A rapid and sensitive method for the quantitation of microgram quantities of protein utilizing the principle of protein-dye binding. Anal Biochem 72: 248–254. 942051 10.1016/0003-2697(76)90527-3

[50] Boffi A, Das TK, Della Longa S, Spagnuolo C, Rousseau DL (1999) Pentacoordinate hemin derivatives in sodium dodecyl sulfate micelles: model systems for the assignment of the fifth ligand in ferric heme proteins. Biophys J 77: 1143–1149. 10423459 10.1016/S0006-3495(99)76965-1 PMC1300405

[51] Kamal JK, Behere DV (2002) Spectroscopic studies on human serum albumin and methemalbumin: optical, steady-state, and picosecond time-resolved fluorescence studies, and kinetics of substrate oxidation by methemalbumin. J Biol Inorg Chem 7: 273–283. 11935351 10.1007/s007750100294

[52] Antonini E, Brunori M (1971) Hemoglobin and Myoglobin in their Reactions with Ligands, North Holland Publishing Co., Amsterdam and London.

[53] Coletta M, Angeletti M, De Sanctis G, Cerroni L, Giardina B, et al (1996) Kinetic evidence for the existence of a rate-limiting step in the reaction of ferric hemoproteins with anionic ligands. Eur J Biochem 235: 49–53. 8631366 10.1111/j.1432-1033.1996.00049.x

[54] Ascenzi P, Fasano M (2009) Serum heme-albumin: an allosteric protein. IUBMB Life 61: 1118–1122. 19946891 10.1002/iub.263

[55] Ascenzi P, Fasano M (2010) Allostery in a monomeric protein: the case of human serum albumin. Biophys Chem 148: 16–22. 20346571 10.1016/j.bpc.2010.03.001

[56] Spiro TG, Wasbotten IH (2005) CO as a vibrational probe of heme protein active sites. J Inorg Biochem 99: 34–44. 15598489 10.1016/j.jinorgbio.2004.09.026

[57] Ray GB, Li XY, Ibers JA, Sessler JL, Spiro TG (1994) How far can proteins bend the FeCO unit? Distal polar and steric effects in heme proteins and models. J Am Chem Soc 116: 162–176.

[58] Vogel KM, Kozlowski PM, Zgierski MZ, Spiro TG (2000) Role of the axial ligand in hemeνCO backbonding; DFT analysis of vibrational data. Inorg Chim Acta 297: 11–17.

[59] Fitos I, Visy J, Simonyi M, Hermansson J (1999) Stereoselective allosteric binding interaction on human serum albumin between ibuprofen and lorazepam acetate. Chirality 11: 115–120. 9951402 10.1002/(SICI)1520-636X(1999)11:2<115::AID-CHIR6>3.0.CO;2-R

[60] Bertucci C, Domenici E (2002) Reversible and covalent binding of drugs to human serum albumin: methodological approaches and physiological relevance. Curr Med Chem 9: 1463–1481. 12173977 10.2174/0929867023369673

[61] Fitos I, Visy J, Kardos J (2002) Stereoselective kinetics of warfarin binding to human serum albumin: effect of an allosteric interaction. Chirality 14: 442–448. 11984760 10.1002/chir.10113

[62] Kragh-Hansen U, Chuang VT, Otagiri M (2002) Practical aspects of the ligand-binding and enzymatic properties of human serum albumin. Biol Pharm Bull 25, 695–704. 10.1248/bpb.25.695 12081132

[63] Ascenzi P, Bocedi A, Notari S, Menegatti E, Fasano M (2005) Heme impairs allosterically drug binding to human serum albumin Sudlow’s site I. . Biochem Biophys Res Commun 334: 481–486. 16004963 10.1016/j.bbrc.2005.06.127

[64] Ahmad E, Rabbani G, Zaidi N, Singh S, Rehan M, et al (2011) Stereo-selectivity of human serum albumin to enantiomeric and isoelectronic pollutants dissected by spectroscopy, calorimetry and bioinformatics. PLoS One 6: e26186. 22073150 10.1371/journal.pone.0026186 PMC3206814

[65] Ahmad E, Rabbani G, Zaidi N, Ahmad B, Khan RH (2012) Pollutant-induced modulation in conformation and β-lactamase activity of human serum albumin. PLoS One 7: e38372. 22685563 10.1371/journal.pone.0038372 PMC3369883

[66] Gullotta F, di Masi A, Ascenzi P (2012) Carbon monoxide: an unusual drug. IUBMB Life 64: 378–386. 22431507 10.1002/iub.1015

[67] Gullotta F, di Masi A, Coletta M, Ascenzi P (2012) CO metabolism, sensing, and signaling. Biofactors 38: 1–13. 22213392 10.1002/biof.192

[68] Adam SS, McDuffie JR, Ortel TL, Willing JW Jr (2012) Comparative effectiveness of Warfarin and new oral anticoagulants for the management of atrial fibrillation and venous thromboembolism. A systematic review. Ann Intern Med, in the press. 10.7326/0003-4819-157-10-201211200-00532 22928173

[69] Coletta M, Ascenzi P, Traylor TG, Brunori M (1985) Kinetics of carbon monoxide binding to monomeric hemoproteins. Role of the proximal histidine. J Biol Chem 260: 4151–4155. 3980472

[70] Ciaccio C, Coletta A, De Sanctis G, Marini S, Coletta M (2008) Cooperativity and allostery in haemoglobin function. IUBMB life 60: 112–123. 18380000 10.1002/iub.6

