# Supplementary material for: Reciprocal Allosteric Modulation of Carbon Monoxide and Warfarin Binding to Ferrous Human Serum Heme-Albumin
Source: PLoS One. 2013 Mar 21;8(3):e58842. doi: 10.1371/journal.pone.0058842 (PMC3605432; doi:10.1371/journal.pone.0058842)
Supplement: Material S1 — Supplementary materials. (DOC) [file pone.0058842.s002.doc]

**Supplementary Material**

**RECIPROCAL ALLOSTERIC MODULATION OF CARBON MONOXIDE AND WARFARIN BINDING TO FERROUS HUMAN SERUM HEME-ALBUMIN**

As previously reported, for CO association three observed rate constants (*i.e.*, ^1^*k_obsCO_*, ^2^*k_obsCO_* and ^3^*k_obsCO_*) occur, where (*i*) ^1^*k_obsCO_*, referring to a reaction involving about 80% of the total observed absorption change (see Fig. S1A), displays a bimolecular behaviour and it has been analysed according to eq. (4) (see Fig. S1B), (*ii*) ^2^*k_obsCO_*, amounting to about 10% of the total observed absorption change (see Fig. S1A), shows a concentration dependence only at relatively low CO concentrations with a levelling off at higher CO concentrations (see Fig. S1B) (thus suggesting the existence of a rate-limiting step) and it has been analysed according to eq. (5a), and (*iii*) ^3^*k_obsCO_*, amounting to about 10% of the total observed absorption change (see Fig. S1A), displays a concentration-independent behaviour, indicating that this signal refers to either a slow conformational change or else to a CO binding process with a very slow rate-limiting event [45].

Unlike the CO association kinetics, CO dissociation from HSA-heme-Fe(II)-CO is characterized by a biphasic kinetic pattern (Fig. S1C), as from the analysis according to eq. (3), suggesting that only two populations of HSA-heme-Fe(II)-CO are sufficient to account for the observed behaviour. The rate constants of the two species differ by one order of magnitude (*i.e.*, ^1^*k_offCO_* = 0.26±0.04 s^-1^, amounting to about 70% of the total observed absorption change, and ^2^*k_offCO_* = 0.029±0.004 s^-1^, amounting to about 30% of the total observed absorption change) (see Table 1). Therefore, the energy barrier for the CO detachment is quite marked between the two forms, the 10-fold difference of *k_offCO_* amounting to an activation free energy change of about 4 kJ/mol.

It is interesting to point out that a previous investigation on CO binding by HSA-heme-Fe(II) [1S], employing laser photolysis, observed a biphasic rebinding. This result is in keeping with what reported by Cao and co-workers [45] and in the present study, considering that in the laser photolysis the experiment starts with HSA-heme-Fe(II)-CO (for which we observe two populations as well, see above) and CO rebinding occurs before the slow re-equilibration of unliganded HSA-heme-Fe(II) species takes place.

The independent determination of the CO dissociation rate constant(s), a parameter which affects also the determination of the CO binding rate constant (see eqs. (4) and (5a)), allowed to fit the CO-dependence of ^1^*k_obsCO_* and of ^2^*k_obsCO_* in a more quantitative way. Thus, the two continuous lines in Fig. S1B indeed refer to non-linear least-squares fitting of ^1^*k_obsCO_* according to eq. (4), imposing ^1^*k_offCO_* = 0.029±0.004 s^-1^, and to a non-linear least-squares fitting of ^2^*k_obsCO_* according to eq. (5a), imposing ^1^*k_offCO_* = 0.26±0.04 s^-1^, respectively (see Table 1).

**REFERENCES**

[1S] Marden MC, Hazard ES, Leclerc L, Gibson QH (1989) Flash photolysis of the serum albumin-heme-CO complex. Biochemistry 28: 4422-4426.
